# Evaluating predictive performance, validity, and applicability of machine learning models for predicting HIV treatment interruption: a systematic review

**DOI:** 10.1186/s44263-025-00184-4

**Published:** 2025-07-24

**Authors:** Williams Kwarah, Frances Baaba da-Costa Vroom, Duah Dwomoh, Samuel Bosomprah

**Affiliations:** 1 Department of Biostatistics, School of Public Health, University of Ghana, Accra, Ghana; 2 United States Agency for International Development (USAID), Ghana Mission, Accra, Ghana

**Keywords:** HIV treatment interruption, Machine learning, Predictive modeling

## Abstract

**Background:**

HIV treatment interruption remains a significant barrier to achieving global HIV/AIDS control goals. Machine learning (ML) models offer potential for predicting treatment interruption by leveraging large clinical data. Understanding how these models were developed, validated, and applied remains essential for advancing research.

**Methods:**

We searched databases including the PubMed, BMC, Cochrane Library, Scopus, ScienceDirect, Lancet, and Google Scholar, for studies published in English from 1990 to September 2024. Search terms covered HIV, machine learning, treatment interruption, and loss to follow-up. Articles were screened and reviewed independently, and data were extracted using the CHecklist for critical Appraisal and data extraction for systematic Reviews of prediction Modelling Studies (CHARMS) tool. Risk of bias was assessed with Prediction model Risk Of Bias Assessment Tool (PROBAST). The Preferred Reporting Items for Systematic reviews and Meta-analysis (PRISMA) guidelines were followed throughout.

**Results:**

Out of 116,672 records, 9 studies met the inclusion criteria and reported 12 ML models. Random Forest, XGBoost, and AdaBoost were predominant models (91.7%). Internal validation was performed in all models, but only two models included external validation. Performance varied, with a mean area under the receiver operating characteristic curve (AUC-ROC) of 0.668 (standard deviation (SD) = 0.066), indicating moderate discrimination. About 75% of models showed a high risk of bias due to inadequate handling of missing data, lack of calibration, and the absence of decision curve analysis (DCA).

**Conclusions:**

ML models show promise for predicting HIV treatment interruption, particularly in resource-limited settings. Future research should prioritize external validation, robust missing data handling, and decision curve analysis and include sociocultural predictors to improve model robustness.

**Systematic review registration:**

PROSPERO CRD42024578109.

**Supplementary Information:**

The online version contains supplementary material available at 10.1186/s44263-025-00184-4.

## Background

Human immunodeficiency virus (HIV) treatment interruption poses a significant challenge to global efforts in the HIV/AIDS epidemic response. In 2022, an estimated 39 million people were living with HIV (PLHIV) globally, with an estimated 1.3 million new infections and 630,000 deaths reported [1]. The burden of HIV infection is disproportionately high in sub-Saharan Africa, Asia, and the Pacific, which together account for about 88% of all cases [2]. Despite the availability of antiretroviral therapy (ART), which has dramatically reduced the progression of HIV to AIDS and decreased AIDS-related mortality, many individuals living with HIV struggle to maintain consistent adherence to their treatment regimen [3, 4]. It has been estimated that only 46% to 85% of patients continue to stay on ART 2 years after initiation [5, 6]. This lack of adherence is particularly concerning given that when left untreated, HIV weakens the immune system and can lead to life-threatening complications [4]. People who stay in treatment are economically viable and productive to their families and the community [7]. Interrupting HIV treatment may result in viral rebound, deterioration of the immune system, heightened transmission risk, and the development of drug resistance, thereby compromising both individual health and community prevention initiatives. The situation places significant pressure on healthcare systems and compromises public health initiatives [8–11].

Improving ART adherence is critical to achieving global HIV/AIDS control goals. While current strategies to address treatment interruption primarily focus on re-engaging patients after missed doses [12, 13], these reactive measures often fall short of preventing the associated health risks and potential for increased transmission. The ability to predict treatment interruptions before they occur could revolutionize HIV care by enabling healthcare providers to implement targeted and proactive interventions that keep patients on therapy, thus enhancing their chances of achieving and sustaining viral suppression. Machine learning (ML) and artificial intelligence (AI) offer powerful tools for developing such predictive models due to their capacity to dynamically analyze large, complex datasets and uncover patterns that traditional methods might miss [14–18]. Despite the promise of these technologies, there remains a significant evidence gap in their application to HIV treatment adherence, particularly in low-resource settings where the burden of the disease is greatest. Addressing this gap through systematic evaluation of existing predictive models is crucial for advancing the use of ML and AI in HIV care. This can lead to more effective and personalized treatment strategies that can help meet the ambitious Joint United Nations Programme on HIV/AIDS (UNAIDS) 95-95-95 targets by 2030 [2].

This systematic review aimed to evaluate the effectiveness of machine learning-based predictive models in forecasting HIV treatment interruptions. Specifically, the review (1) identified the types of predictive models previously developed, (2) assessed their accuracy and applicability in various settings, and (3) determined which models have been validated and how they performed in different populations. The impact of this review could provide insights that can guide the integration of advanced predictive technologies into HIV care programs, potentially improving patient retention, optimizing treatment outcomes, and supporting global efforts to eliminate HIV as a public health threat by 2030.

## Methods

### Search strategy

We searched multiple electronic databases, including Scopus, PubMed, *The Lancet*, BioMed Central (BMC) Public Health, ScienceDirect, Google Scholar, and Cochrane Library. Our search covered publications from January 1990 to September 2024. We searched using a combination of Medical Subject Headings (MeSH) and free-text terms. The key terms included “HIV,” “Human Immunodeficiency Virus,” “AIDS,” and “Acquired Immunodeficiency Syndrome” for HIV-related concepts; “Machine Learning,” “ML,” “Artificial Intelligence,” “AI,” “Neural Networks,” and “Predictive Modeling” for machine learning concepts; and “Treatment Interruption,” “Loss to Follow-Up “Non-adherence,” “Default,” and “Treatment Discontinuation” for treatment adherence concepts. These terms were combined using Boolean operators (AND, OR) to ensure a broad and inclusive search. Details of the search strategy for each database is provided in Additional File 1: Search Strategy.

### Eligibility criteria

We applied specific eligibility criteria to select studies for inclusion. Eligible studies focused on developing or validating prediction models for HIV treatment interruption at the individual level using machine learning methods. We only included studies published in English. We included studies that focused on HIV treatment interruption defined as missing a scheduled clinic or pharmacy appointment by at least 28 days. We excluded studies that identified predictors without focusing on prediction models and studies lacking full-text availability. Reviews, commentaries, conference abstracts, letters, reports, and opinions were excluded. In addition to database searches, we manually reviewed the reference lists of the included studies to identify additional relevant articles. To capture recent and unpublished research, we searched preprint servers such as bioRxiv, medRxiv, and arXiv. The corresponding authors of the included articles were emailed to seek further information and clarity. The search strategy was carefully documented (Additional File 1), and articles were managed using *Zotero 6.0.37* reference management software, a project of Digital Scholar [19]. The Preferred Reporting Items for Systematic reviews and Meta-Analysis (PRISMA) statements [20] (Additional File 2) and the conduct of systematic reviews [21] guided the review. A protocol for this review was registered on PROSPERO CRD42024578109.

### Selection process

Article selection was conducted in multiple stages to ensure that only studies meeting the predefined inclusion criteria were included. Initially, two independent reviewers (W. K. and G. J. P.I.) screened the titles and abstracts of all records retrieved from the database searches to identify potentially relevant studies. We resolved any disagreements between reviewers during the article selection process through discussion, and a third reviewer (N.Z.) was available to adjudicate unresolved disputes. To enhance the rigor of the selection process, systematic review software *Distiller SR 2.35* developed by DistillerSR Incorporated [22] was used to assist in the identification and removal of duplicate records before the screening began.

### Data extraction

Two independent reviewers (W K. and G. J. P.I.) extracted data from the selected studies to ensure accuracy and consistency. Each reviewer independently extracted data, using the standardized CHecklist for critical Appraisal and data extraction for systematic Reviews of prediction Modelling Studies (CHARMS) tool [23, 24]. CHARMS was developed for systematic reviews of prognostic or diagnostic prediction models without external validation, with external validation, or external prediction model validation with or without model updating. The data collected included the data sources, study characteristics, details of the predictive models, outcomes, and performance metrics [24]. We resolved any disagreements between reviewers during the data extraction process through discussion, and a third reviewer (N.Z.) was available to adjudicate unresolved disputes. The reviewers manually extracted all data and then cross-verified it to maintain the integrity of the data collection process. A consolidated final completed CHARMS tool was compiled for this review.

### Risk of bias and applicability assessment

We used the Prediction Model Risk of Bias Assessment Tool (PROBAST) [25] to assess the risk of bias (ROB) and applicability in the included studies. The PROBAST was designed to evaluate the risk of bias and applicability in prediction model studies. The tool evaluated four key domains: participants, predictors, outcomes, and analysis. There were two questions on participants, three questions on predictors, six questions about outcomes, and nine questions linked to the statistical analysis. Responses to these questions were either “yes,” “probably yes,” “probably no,” “no,” or “no information.” The ROB was classified as either low, high, or unclear based on the responses within these domains. A domain was classified as high risk if it included at least one question that has been answered with either “no” or “probably no,” low risk if all the questions indicated as “yes” or “probably yes,” and unclear if there is no information in the responses. If all domains were assessed as having a low risk, then the overall risk of bias was classified as low. However, if at least one domain was determined to have a high risk, then the overall risk of bias was classified as high. If there was a recognized concern for bias in at least one area and the level of concern was low for all other domains, it was classified as having a moderate level of concern for bias. Two reviewers (W. K. and G. J. P.I.) independently evaluated the risk of bias in each included study. When the reviewers disagreed on the risk-of-bias judgment, the discrepancies were discussed to reach a consensus. If the disagreement persisted, a third reviewer (N.Z.) was consulted to decide. Similarly, model applicability was assessed in the first three domains — participants, predictors, and outcome for each model. Model applicability was rated low concern, high concern, or unclear concern based on a defined rubric [25]. If there were low concerns regarding applicability for all domains, the prediction model evaluation was judged to have low concerns regarding applicability. If there were high concerns regarding applicability for at least one domain, the prediction model evaluation was judged to have high concerns regarding applicability. If there were unclear concerns (but no “high concern”) regarding applicability for at least one domain, the prediction model evaluation was judged to have unclear concerns regarding applicability overall. We conducted all evaluations manually and documented the results of the risk-of-bias assessments and applicability in detail, with summary judgments presented in the form of charts to facilitate a clear understanding of the quality and reliability of the included studies.

### Synthesis and analysis

We tabulated the results of individual studies to provide a clear and organized presentation of the key findings. This included details such as study characteristics, model performance metrics (e.g., area under the receiver-operating characteristic curve, calibration statistics), and risk-of-bias assessments. We used visual displays, including charts to enhance the clarity of the results and to facilitate the comparison of study outcomes. For the synthesis of results, we used a narrative synthesis approach due to the anticipated heterogeneity of the included studies, particularly in terms of model types, outcome measures, and study populations. This approach allowed us to systematically describe and compare the predictive models, highlighting common themes and differences among the studies. We did not perform a meta-analysis because there were insufficient external validation studies of the same index model to justify a quantitative synthesis [21]. The synthesis followed guidelines from the Transparent Reporting of a Multivariable prediction model for Individual Prognosis Or Diagnosis (TRIPOD) statement [26], CHARMS checklist [24], and PROBAST [25].

## Results

### Characteristics of included studies

**Fig. 1:**
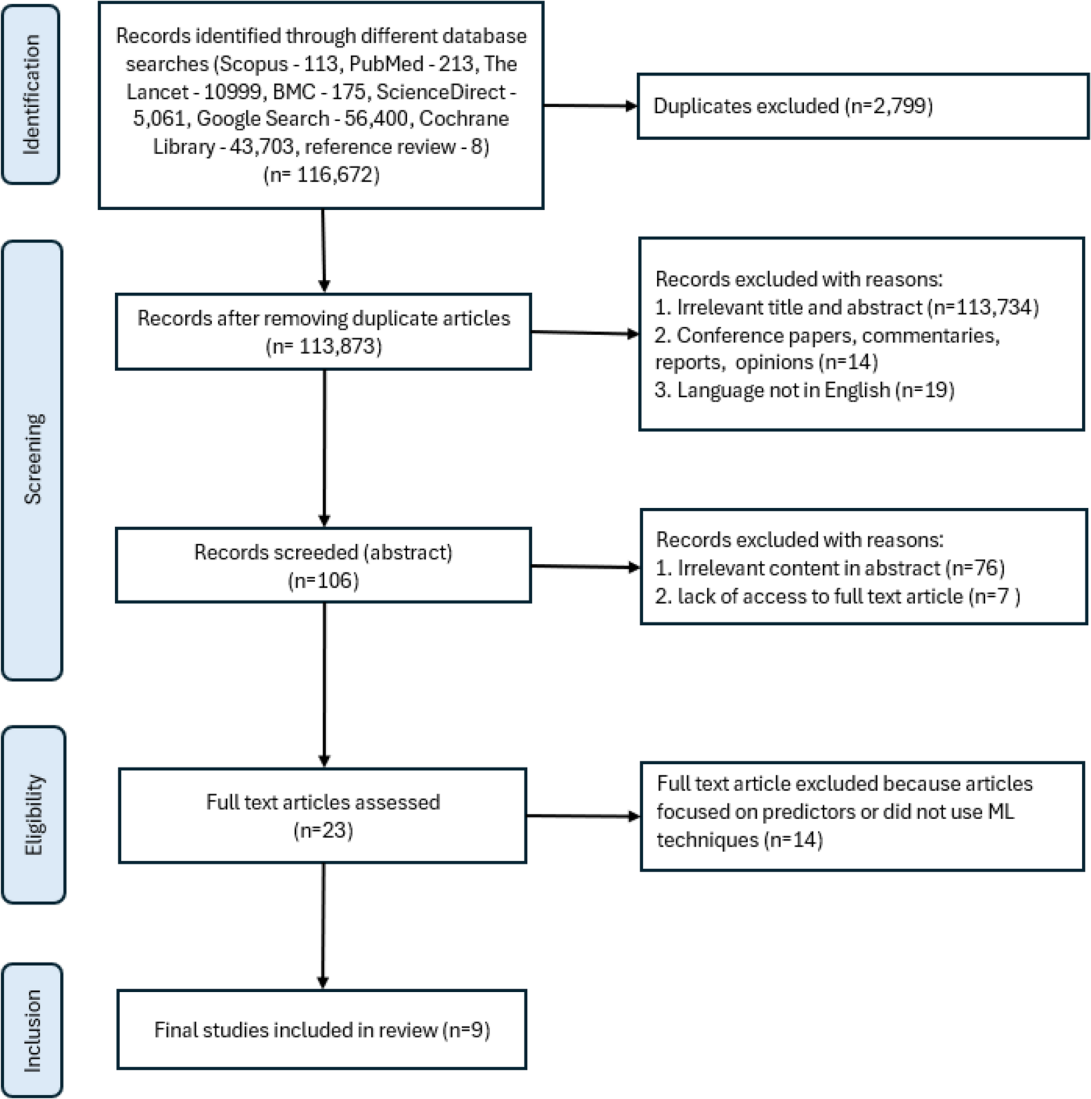
PRISMA flow of article selection

Our search identified 116,672 studies, of which 9 met the inclusion criteria (Fig. 1). Seven of these studies focused on developing predictive models [27–33], while two included both model development and validation [34, 35]. Six studies were conducted in Africa [27–30, 33, 34], of which three were in South Africa, one in Tanzania, and one combining data from Nigeria and Mozambique. The remaining three studies were in the United States of America (USA) [31, 32, 35] (Table 1). These studies were published between 2018 and 2024, with the majority published in 2023 and 2022. Seven studies were conducted in public healthcare facilities, while two were conducted in university clinics. Seven studies relied on retrospective cohort data, while two used existing registries (Table 1). Heterogeneity was not explored as only three models were externally validated.

**Table 1:** Characteristics of the included studies

Author, Year	Study Design	ML Technique	Enrolment period	Study setting	Study region	Age of participants	Female	Male	Treatment Interruption
1 - Matthew-David Ogbechie, 2023 [30]	Retrospective cohort	XGBoost	January 2005 - February 2021	Health facility	Nigeria		91982 (67.3)	44765 (32.7)	56581 (41.5)
2 - Esra, Rachel, 2023 [34]	Existing registry	AdaBoost	January 1, 2017 - March 31, 2020	Public health facilities	South Africa	33 (27-41)	172170 (65)	92707 (35)	260467 (11.9)
		CatBoost	January 1, 2017 - October 1, 2018	Public health facilities	South Africa	33 (27-41)	172170 (65)	92707 (35)	
3 - Stockman, Jeni , 2022 [33]	Retrospective cohort	Random Forest	January 1, 2010 - November 28, 2019	Public Sector ART clinics	Mozambique	47.3 (13.6)			
		XGBoost		Public Sector ART clinics	Nigeria	47.3 (13.6)			
4 - Arthi, Ramachandran, 2020 [32]	Retrospective cohort	Random Forest	January 1, 2008 to May 31, 2015	University of Chicago HIV care clinic	USA	47.3 (13.6)	314 (44%)	399 (56%)	
		Decision Trees		University of Chicago HIV care clinic	USA	47.3 (13.6)	314 (44%)	399 (56%)	
5 - Brian W. Pence, 2018 [31]	Retrospective cohort	Logistic Regression	2002 - 2015	US-based HIV primary care clinics	USA	46 (39 - 52)	1660 (16)	8714 (84)	17957 (17)
6 - Mhairi, Maskew, 2022 [28]	Retrospective cohort	AdaBoost	January 2016 - December 2018	Public Sector HIV care facilities	South Africa	39 (31 - 49)	311,945 (70%)	133,690 (30%)	
7 - Mhairi, Maskew, 2024 [29]	Retrospective cohort	AdaBoost	January 2016 - December 2018	Public Sector HIV care facilities	South Africa	39 (27 - 49)	315124 (68)	148294 (32)	
8 - Joseph A Mason, 2023 [35]	Existing registry	Random Forest	Jan 21 - March 30, 2022	Hospital in a university	USA				
9 - Carolyn A Fahey, 2022 [27]	Retrospective cohort	Decision Trees	2018	HIV care center	Tanzania	36 (10)	113 (63.5)	65 (36.5)	

### Model performance metrics

Model performance is often measured using different metrics such as overall performance measures, discrimination, calibration, and (re)classification. Discrimination assesses the model’s capacity to differentiate between individuals who have and do not have the outcome. The c-statistic, which is equivalent to the area under the curve of a receiver operating characteristic curve (AUC-ROC), is frequently used to assess discrimination. Other classification measures such as sensitivity, specificity, negative predictive value (NPV), positive predictive value (PPV), and F1 score are often used to assess model discrimination. Calibration measures how well the predicted risks and observed outcomes match and is often assessed using graphical comparison of the observed and predicted event rates. Formal statistical tests such as the Hosmer–Lemeshow test for logistic regression are commonly used in conjunction with calibration plots.

Among the 9 studies selected, a total of 12 machine learning models were reported, with 9 focused on model development and 3 on model validation (Table 2). The median sample size across studies was 136,415 (interquartile range: 178–450,000), though 1 model was developed using a sample size of less than 1000 participants. On average, 15 predictors (standard deviation (SD) = 4.0) were included in the final models. Ensemble learning techniques were the most frequently used algorithms, accounting for 92% of the total models. These included random forest (three models), Adaptive Boosting (AdaBoost, three models), Extreme Gradient Boosting (XGBoost, two models), Decision Trees (two models), and Categorical Boosting (CatBoost, one model) (Table 2). Logistic regression was used in only one model.

**Table 2:** Summary of model performance metrics using the CHARMS checklist

Author, Year	Modelling method	Sample size	Events n (%)	No predictors Candidates	Final predictors	EPV or EPP	Selection of candidate predictors	Selection of final predictors	Number (%) and handling of missng data	Type of validation	Performance measures
1 - Matthew-David Ogbechie, 2023 [30]	XGBoost	136,747	56581 (41.4)	13	13	4352.4	Based on prior knowledge	Pre-specified model (not selection)	n (%): Unkown Method: Knn Imputation	Int: Cross-validation and random split data Ext : None	Calibration measures: Not evaluated Discrimination measures : Accuracy 0.85 (0.85 - 0.86), Sensitivity - 0.81; Specificity - 0.88; PPV - 0.83; NPV - 0.87; Kappa 0.70 Overall measures: Not evaluated
2 - Esra, Rachel, 2023 [34]	AdaBoost	264,877	35985 (13.6)	13	13	2768.1	Based on prior knowledge	Recursive feature elimination	n (%): 1509 (0.6) Method: Single imputation	Int: Random split data Ext : Different setting	Calibration measures: F1 Score (0.288, 0.286 - 0.290) Discrimination measures : C-Statistic / AUC graph / Sensitivity (0.608, 0.604 - 0.611), specificity (0.647, 0.646 - 0.648), ppv (0.189, 0.187 - 0.190), npv (0.924, 0.924 - 0.925) Overall measures: Not evaluated
3 - Esra, Rachel, 2023 [34]	CatBoost	136,082	35985 (26.4)	13	13	2768.1	Based on prior knowledge	Recursive feature elimination	n (%): 1509 (1.1) Method: Single imputation	Int: Random split data Ext : Different setting	Calibration measures: F1 Core (0.299, 0.297 - 0.301) Discrimination measures : C-Statistic / AUC graph / Sensitivity (0.646, 0.642 - 0.649), specificity (0.646, 0.645 - 0.648), ppv (0.195, 0.193 - 0.196), npv (0.932, 0.931 - 0.933) Overall measures: Not evaluated
4 - Stockman, Jeni , 2022 [33]	Random Forest	360,000		70	12	Unknown	Other	No information	n (%): Unkown Method: Missing values excluded in analysis	Int: Cross-validation Ext : No information	Calibration measures: Not evaluated Discrimination measures : C-Statistic / AUC-PR, MCC (0.45) Overall measures: Not evaluated
5 - Stockman, Jeni , 2022 [33]	XGBoost	450,000		70	12	Unknown	Other	No information	n (%): Unkown Method: Missing values excluded in analysis	Int: Temporal cross-validation Ext : No information	Calibration measures: Not evaluated Discrimination measures : C-Statistic / AUC-PR, MCC(0.37) Overall measures: Not evaluated
6 - Arthi, Ramachandran, 2020 [32]	Random Forest	11,445	1373 (12.0)	1000	20	1.4	Based on prior knowledge	Other	n (%): Unkown Method: Single imputation	Int: Temporal cross-validation Ext : No information	Calibration measures: Not evaluated Discrimination measures : PPV (24.5, SD = 0.01) Overall measures: Not evaluated
7 - Arthi, Ramachandran, 2020 [32]	Decision Trees	11,445	1373 (12.0)	800	20	1.7	Based on prior knowledge	Other	n (%): Unkown Method: Single imputation	Int: Random split data Ext : No information	Calibration measures: Not evaluated Discrimination measures : PPV (15.5, 0.04) Overall measures: Not evaluated
8 - Brian W. Pence, 2018 [31]	Logistic regression	105,628	17957 (17.0)	14	14	1282.6	Based on prior knowledge	Pre-specified model (not selection)	n (%): Unkown Method: No information	Int: Cross-validation Ext : No information	Calibration measures: Not evaluated Discrimination measures : C-Statistic / AUC graph / Sensitivity (0.74, 0.70 - 0.78), Specificity (0.54, 0.44 - 0.64) Overall measures: Not evaluated
9 - Mhairi, Maskew, 2022 [28]	AdaBoost	1,399,145	146881 (10.5)	75	20	1958.4	Other	Feature selection using random forest	n (%): Unkown Method: Other	Int: Random split data Ext : No information	Calibration measures: F1 Score (0.29) Discrimination measures : C-Statistic / AUC graph / Accuracy (0.786), sensitivity (0.406), specificity (0.83), npv (0.92), ppv (0.22)Disc : C-Statistic / AUC graph / Accuracy (0.786), sensitivity (0.406), specificity (0.83), npv (0.92), ppv (0.22) Overall measures: Not evaluated
10 - Mhairi, Maskew, 2024 [29]	AdaBoost	3,264,671	146881 (4.5)	11	10	13352.8	No information	No information	n (%): Unkown Method: No information	Int: Random split data Ext : No information	Calibration measures: Not evaluated Discrimination measures : C-Statistic / Accuracy = 0.63, Specificity = 0.52, specificity =0.64, ppv = 0.19, NPV = 0.89 Overall measures: Not evaluated
11 - Joseph A Mason, 2023 [35]	Random Forest	331	0 (0.0)	11	11	0	Based on prior knowledge	No information	n (%): Unkown Method: No information	Int: Random split data Ext : Different dataset and provider feedback	Calibration measures: Not evaluated Discrimination measures : C-Statistic / AUC graph Overall measures: Not evaluated
12 - Carolyn A Fahey, 2022 [27]	Decision Trees	178	72 (40.4)	22	22	3.3	Based on prior knowledge	No information	n (%): 0 (0.0) Method: Other	Int: Cross-validation and random split data Ext : Unclear	Calibration measures: Not evaluated Discrimination measures : C-Statistic / Accuracy (0.723) Overall measures: Not evaluated

*EPV* Events per variable, *EPP* Events per predictor, *PPV* Positive Predictive Value, *NPV* Negative Predictive Value, *AUC-PR* Area Under the Precision Recall Curve, *MCC* Matthews Correlation Coefficient

Model performance was primarily assessed using the c-statistic or area under the receiver operating characteristic curve (AUC), with an average AUC of 0.668 (*SD*: 0.07). Some models also reported additional metrics, including accuracy, sensitivity, specificity, negative predictive value (NPV), and positive predictive value (PPV) (Table 2). Notably, two models reported only PPV, while another two reported the Mathews correlation coefficient. Model calibration methods were used in just three models, which reported an average F1 score of 0.292 (*SD*: 0.01) alongside the AUC. None of the studies used decision curve analysis (DCA) to assess clinical value and implications, a significant limitation in evaluating the practical utility of the models. DCA is essential for assessing a model’s clinical relevance by weighing the benefits and risks at different decision thresholds, rendering its exclusion a significant constraint [36]. DCA are essential metrics that enhance calibration and discrimination measures in machine learning models [37] and help in incorporating the clinical consequences of using a model. Besides conducting DCA, net benefit analysis is an alternative measure to assess the applicability of models in real-life situations. However, one study addressed model utility by gathering feedback from healthcare workers. Additional information is provided in Additional File 3: Model Characteristics Tab.

### Risk-of-bias assessment

**Fig. 2:**
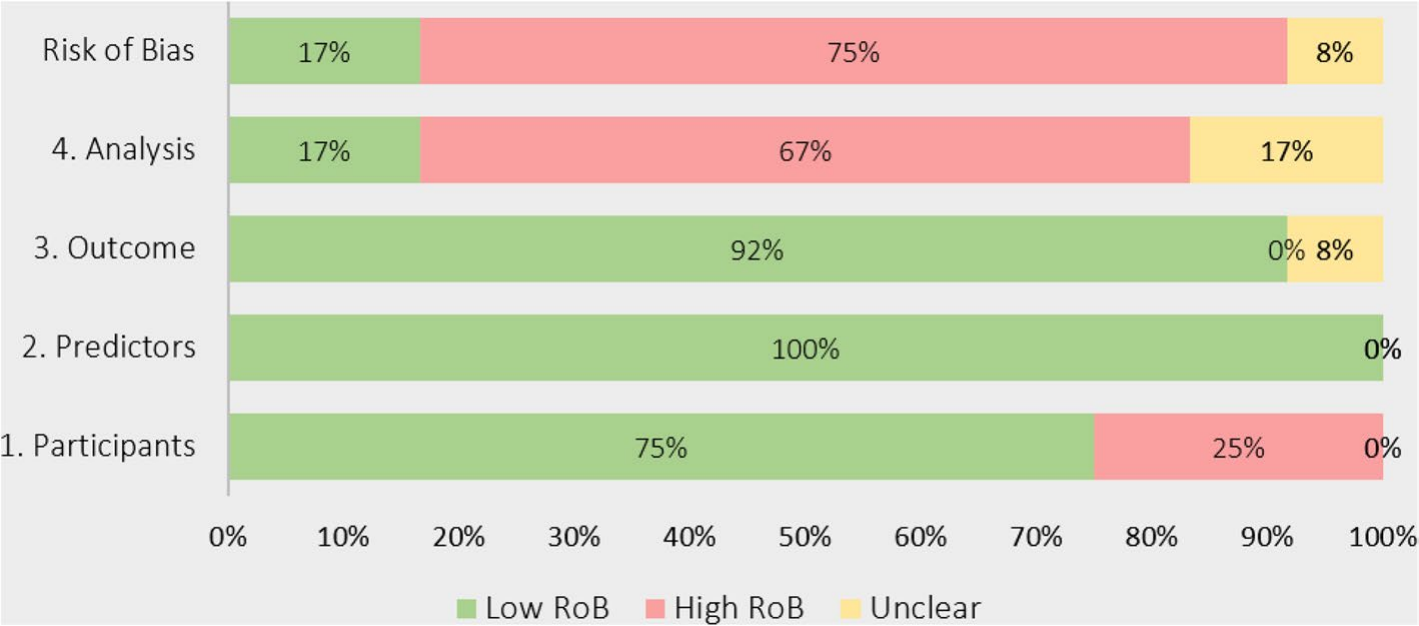
Summary of risk-of-bias assessment

We reported the risk-of-bias assessment for the 12 models using the PROBAST tool (Fig. 2). Of these, nine models (75.0%) were rated as having a high risk of bias, two models (16.7%) were rated low risk, and one model (8.3%) had an unclear risk of bias. A notable majority (58.3%) expressed high risk in the statistical analysis domain. For example, nearly half of the models failed to report how missing data was handled, and 10 models (83.3%) did not disclose the extent of missing data. Furthermore, only three models (25.0%) provided details on calibration measures, which are important for ensuring the reliability of predictions. None of the studies reported DCA or other methods to assess clinical utility, highlighting a critical gap in evaluating the practical application of these models. Additional details on the risk-of-bias analysis are provided in the supplementary material provided in Additional File 3: PROBAST summary tables.

### Applicability assessment

**Fig. 3:**
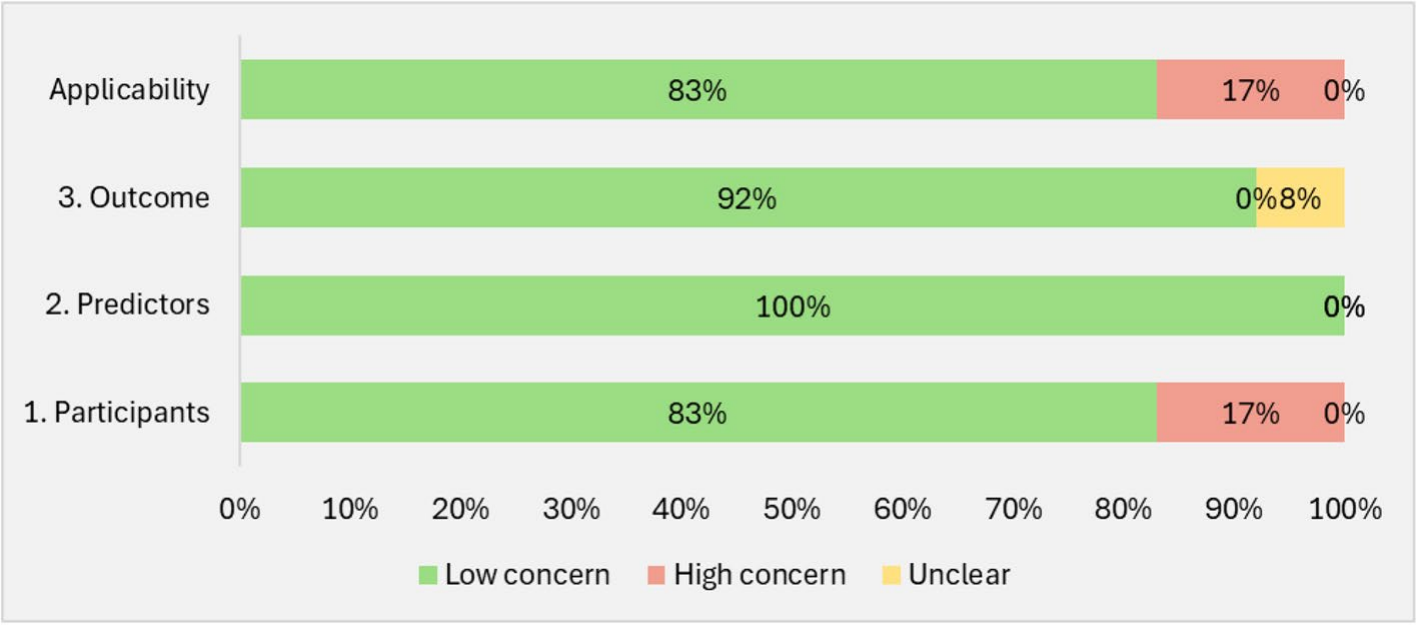
Summary of applicability assessment

We evaluated the applicability of the models for use in the intended population and primary healthcare settings. Overall, 83% of the models were rated as low concern, indicating their suitability for primary healthcare use. However, 17% were rated as high concern, reflecting limitations in certain aspects of model development (Fig. 3). Predictors were rated as low concern, suggesting that the included predictors were relevant to the target population and routinely collected in clinical settings. Similarly, the outcome domain was rated as low concern in 92% of the models, while 8% were marked as unclear due to insufficient reporting of key details.

### Model validation

All 12 models reported internal validation. These included random sample split (6), cross-validation (4), and a combination of random sample split and cross-validation (2) (Table 2). Three models were externally validated, but only two reported discrimination measures, with an average F1 score of 0.2935, alongside c-statistic (AUC) values. These validations were done using datasets received from registries of people living with HIV and scheduled for clinical appointments. While sensitivity, specificity, PPV, and NPV were included, one model lacked critical details on eligibility criteria and missing data handling. None of the externally validated models assessed clinical utility. Further details are provided in the supplementary material (Additional File 3: Model characteristics tables).

## Discussions

This review examined 12 machine learning models developed to predict interruptions in HIV treatment, with most relying on advanced ensemble techniques like random forest, AdaBoost, and XGBoost. These models were built using data from large retrospective cohorts, with a median sample size of 120,000 participants, and were validated internally through methods like cross-validation and random sample splitting. The models demonstrated acceptable predictive performance, with an average AUC-ROC of 0.668, and utilized data commonly collected in clinical settings, making them practical for real-world use. For prognostic predictive models, AUC of 0.5–0.7 suggests poor discrimination, and 0.7–0.8 is considered acceptable, 0.8–0.9 excellent, and > 0.9 as outstanding [38, 39]. Although only two models were externally validated, most models showed strong potential for application in primary healthcare, highlighting their promise in improving adherence and supporting HIV care strategies.

Electronic medical records (EMRs) are increasingly prevalent worldwide, including in Africa [40], facilitating the ongoing accumulation of extensive healthcare data and enabling big data analytics [41–46], as well as the application of machine learning and artificial intelligence [44, 47, 48]. Numerous prognostic studies have employed EMR data to create models for predicting individual diagnoses of HIV, healthcare attendance, and viral load suppression [49–51]. The growing utilization of these analytic tools is likely due to the interest in employing predictive models as decision support instruments at the point of care. Moreover, executing focused, high-impact treatments with limited resources in underprivileged healthcare environments is essential [52, 53].

Two-thirds of the research was conducted in Africa, predominantly in South Africa, an area characterized by a high incidence of HIV [54]. This emphasis is praiseworthy, yet it constrains the comprehension of predictive model application in areas with low prevalence. Utilizing data from high-prevalence regions, such as South Africa, offers essential insights into models that help tackle adherence difficulties in analogous circumstances. This emphasis requires careful consideration when extrapolating results to areas with varying healthcare systems and compliance challenges. The research conducted in the USA [31, 32, 35], however limited in number, offered a divergent viewpoint, highlighting the necessity for regionally appropriate models.

The machine learning techniques in our analysis have shown significant potential in forecasting treatment interruption by utilizing routinely gathered clinical data. Ensemble learning methodologies, specifically random forest, AdaBoost, and XGBoost, were significant, collectively representing 91.7% of the models created. Previous studies have demonstrated that ensemble approaches effectively address the complex, nonlinear interactions prevalent in healthcare datasets [55, 56]. These algorithms have achieved above 90% accuracy across many datasets [57, 58]. Ensemble algorithms are beneficial because of their resilience to overfitting and their capacity to handle extensive feature sets. The outcomes of our review correspond with these results. Upon analysis, most models in our study provided the c-statistic (AUC), which evaluates the discriminatory capability of predictive models. The average AUC of 0.668 in our analysis aligns with the findings of Chilamkurthy et al. (2018) who stated that whereas ML models excel at distinguishing different outcomes, their clinical performance criteria, such as accuracy, sensitivity, and specificity, frequently lack efficacy due to unbalanced datasets or inadequate predictor selection often found in healthcare datasets. Other studies have emphasized the need for ML algorithms to employ the AUC as a more effective and superior metric in conjunction with calibration and decision curve analysis for assessing model performance in comparison to accuracy [59].

We discovered in our review that several studies failed to include calibration and clinical efficacy in their reports. Although there are many possible problems in the creation and validation of prediction models, it is essential to disclose calibration measurements, which are vital components of statistical performance [60, 61]. Calibration measures are essential since they guarantee that model prediction probabilities correspond with real probabilities, hence ensuring model dependability. Merely 25% of the research included in our evaluation assessed model calibration. In the absence of calibration, predictive models may provide probabilities that inaccurately reflect actual hazards, hence compromising their therapeutic relevance [62]. We noted significant problems with the ROB in the developed prediction models. Seventy-five percent of the reviewed models were classified as exhibiting a high risk of bias, mostly due to inadequacies in the statistical analysis and data management. Approximately 83.3% of models did not disclose the magnitude of missing data or the methodologies employed to mitigate it, underscoring its significance as a key concern. This conclusion aligns with prior research demonstrating that most predictive model studies do not report their methods for addressing missing data [63]. Missing data is a widespread problem in retrospective healthcare datasets and, if not properly managed, can compromise model performance and integrity [63–65]. Several studies have utilized imputation approaches, precisely predicting missing values to mirror reality, which increases the probability of acquiring high-quality and reusable data [66]. However, if this is not handled appropriately, it can lead to systemic biases and diminish the validity and integrity of models, particularly in datasets utilized in healthcare research [67, 68]. Furthermore, our review observed the lack of decision curve analysis (DCA) in all the studies included. Besides conducting DCA, net benefit analysis is an alternative measure to assess the applicability of models in real-life situations.

The reviewed models show potential for improving HIV treatment interruption predictions; nevertheless, their reliability and applicability in clinical environments are constrained, as shown in the risk-of-bias and applicability results. Overall, an 83% applicability score was achieved for the reviewed models, suggesting their broad appropriateness for the target groups and settings. This result indicates the incorporation of frequently gathered predictors in clinical contexts, including demographic information, adherence records, and clinical indicators, which improve the practicality of applying these models in actual healthcare settings [69]. Ninety-two percent of models assessed the outcome domains as minimal concern; nevertheless, the absence of external validation and decision curve analysis presents serious constraints in the practical use in guiding clinical decisions [62]. For optimal real-world applicability, models must address these deficiencies by integrating external validation across diverse contexts and evaluating clinical significance using methodologies such as DCA, net benefit analysis, or net reclassification improvement assessments. Aligning with clinical processes is crucial for maximizing the efficacy of machine learning in enhancing adherence and minimizing inappropriate treatment exclusion in HIV care. Enhancing future research through stringent reporting standards and robust statistical methodologies, such as those outlined in the TRIPOD recommendations, is essential to mitigate biases and improve the reliability of predictive modeling in HIV care [70].

The results of this review should be interpreted with certain limitations in mind. First, the review included only journal articles published in English with free-text availability, and the search was conducted across a limited number of databases, which may introduce language and publication bias. Excluding studies conducted in other languages besides English presented a potential selection bias. This potentially limits the generalizability of the findings to English-speaking settings. To address potential selection and publication bias stemming from the restricted database search, we supplemented our efforts by conducting backward and forward citation searches in Google Scholar and reviewing article references. Most of these studies were conducted in resource-poor settings, which made it difficult for validation studies to be carried out. It is recommended that in such circumstances, validation studies should be conducted on different datasets or settings.

Future studies should prioritize implementing robust external validation across diverse populations and geographic regions, which is essential to evaluate model performance under varying demographic, clinical, and systemic conditions, ensuring reliability in real-world applications. The inclusion of sociocultural and structural factors in model development should be considered in future research. Also, addressing missing data is critical for enhancing model accuracy and reliability. Future studies should adopt systematic strategies such as multiple imputations or sensitivity analyses and adhere to standardized reporting guidelines like TRIPOD. Finally, incorporating decision curve analysis (DCA) into model assessment is recommended to bridge the gap between statistical performance and practical, real-world impact.

## Conclusions

This study provides key insights into the current state of predictive modeling for HIV treatment interruptions. Machine learning, particularly ensemble learning techniques, is popularly used with retrospective cohort data to address adherence issues in HIV programs, demonstrating moderate accuracy and applicability in primary healthcare settings. However, critical shortcomings, including insufficient calibration reporting, lack of decision curve analysis (DCA), and limited external validation, restrict the models’ clinical utility and generalizability. Predictive modeling holds significant promise in supporting countries to achieve the UNAIDS 95-95-95 targets by advancing equitable access to medications, high treatment retention rates, and achieving widespread viral load suppression.

## Supplementary Information

Additional File 1: Search Strategy (Revised).

Additional File 2: PRISMA 2020 Checklist.

Additional File 3: CHARMS checklist, PROBAST checklist. Study characteristics: Table 1. Characteristics of the studies included in the systematic review. Model characteristics: Table 2: Characteristics of the models included in the systematic review and critical for risk of bias and applicability. PROBAST summary: Table 3: Risk of Bias and applicability assessment. Drop-down lists for CHARMS.

## Data Availability

All data generated or analyzed during this study are part of the supplementary information in the Additional File 3: SUMMARY, CHARMS, and PROBAST tabs.

## Abbreviations

HIV: Human immune virus
AIDS: Acquired immunodeficiency syndrome
PLHIV: People living with HIV
ART: Antiretroviral therapy
ML: Machine learning
AI: Artificial intelligence
UNAIDS: Joint United Nations Programme on HIV/AIDS
PRISMA: Preferred Reporting Items for Systematic reviews and Meta-Analyses
PROSPERO: International Prospective Register of Systematic Reviews
BMC: BioMed Central
MeSH: Medical Subject Headings
CHARMS: CHecklist for critical Appraisal and data extraction for systematic Reviews of prediction Modelling Studies
PROBAST: Prediction model Risk Of Bias Assessment Tool
ROB: Risk of bias
TRIPOD: Transparent Reporting of a multivariable prediction model for Individual Prognosis Or Diagnosis
SD: Standard deviation
XGBoost: Extreme Gradient Boosting
AdaBoost: Adaptive Boosting
CatBoost: Categorical Boosting
AUC-ROC: Area under the receiver operating characteristic curve
AUC-PR: Area under the precision-recall curve
NPV: Negative predictive value
PPV: Positive predictive value
EPV: Events per variable
EPP: Events per predictor
MCC: Mathews correlation coefficient
DCA: Decision curve analysis
EMR: Electronic medical records
