# Supplementary material for: Evaluating predictive performance, validity, and applicability of machine learning models for predicting HIV treatment interruption: a systematic review
Source: BMC Glob Public Health. 2025 Jul 24;3:64. doi: 10.1186/s44263-025-00184-4 (PMC12288308; doi:10.1186/s44263-025-00184-4)
Supplement: Supplementary file 1 — Additional File 1: Search Strategy (Revised). [file 44263_2025_184_MOESM1_ESM.docx]

Additional File 1 Search Strategy (Revised)

| **No.** | **Database** | **Search terms** | **No. articles** |
| --- | --- | --- | --- |
| 1 | Pubmed | ( hiv OR "Human Immunodeficiency Virus" OR aids ) AND ( "Machine Learning" OR "ML" OR "Artificial Intelligence" OR "AI" OR "Neural Networks" OR "Deep Learning" ) AND ( "Treatment Interruption" OR "Loss to Follow-Up" OR "Default" OR "Non-adherence" OR "Interruption in treatment" ) AND ( "Predict*" OR "Forecast*" OR "Model*" ) | 213 |
| 2 | BMC – Public Health | ( hiv OR "Human Immunodeficiency Virus" OR aids ) AND ( "Machine Learning" OR "ML" OR "Artificial Intelligence" OR "AI" OR "Neural Networks" OR "Deep Learning" ) AND ( "Treatment Interruption" OR "Loss to Follow-Up" OR "Default" OR "Non-adherence" OR "Interruption in treatment" ) AND ( "Predict*" OR "Forecast*" OR "Model*" ) | 175 |
| 3 | The Lancet | forecast* OR predict* AND aids OR hiv AND treatment OR interruption OR loss OR ltfu OR iit OR adherence OR non-adherence AND machine AND learning OR ml OR artificial AND intelligence | 10999 |
| 4 | Scopus | forecast* OR predict* AND aids OR hiv AND treatment OR interruption OR loss OR ltfu OR iit OR adherence OR non-adherence AND machine AND learning OR ml OR artificial AND intelligence | 113 |
| 5 | ScienceDirect | predict AND aids/hiv AND treatment AND interruption OR adherence AND machine AND learning | 5061 |
| 6 | Cochrane Library | HIV OR "Human Immunodeficiency Virus" OR "Acquired Immunodeficiency Syndrome" AND "Machine Learning" OR "Artificial Intelligence" OR "Neural Networks" OR "Deep Learning" OR "Predictive Modeling" AND "Treatment Interruption" OR "Loss to Follow-Up" OR Default OR "Non-adherence" AND predict* OR forecast* in Title Abstract Keyword - with Publication Year from 1990 to 2024, with Cochrane Library publication date Between Jan 1990 and Sep 2024, in Trials (Word variations have been searched) | 43703 |
| 7 | Google Scholar | (hiv OR "Human Immunodeficiency Virus" OR aids) AND ( "Machine Learning" OR "ML" OR "Artificial Intelligence" OR "AI" OR "Neural Networks" OR "Deep Learning" ) AND ( "Treatment Interruption" OR "Loss to Follow-Up" OR "Default" OR "Non-adherence" OR "Interruption in treatment" ) AND ( "Predict*" OR "Forecast*" OR "Model*" ) by year 1990-2024 | 56400 |
| 8 | Selected article reference search |  | 8 |
